# Historic Museum Samples Provide Evidence for a Recent Replacement of *Wolbachia* Types in European *Drosophila melanogaster*

**DOI:** 10.1093/molbev/msad258

**Published:** 2023-11-23

**Authors:** Anton Strunov, Sandra Kirchner, Julia Schindelar, Luise Kruckenhauser, Elisabeth Haring, Martin Kapun

**Affiliations:** Center for Anatomy and Cell Biology, Medical University of Vienna, Vienna, Austria; Natural History Museum Vienna, Central Research Laboratories, Vienna, Austria; Natural History Museum Vienna, Central Research Laboratories, Vienna, Austria; Natural History Museum Vienna, Central Research Laboratories, Vienna, Austria; Department for Evolutionary Biology, University of Vienna, Vienna, Austria; Natural History Museum Vienna, Central Research Laboratories, Vienna, Austria; Department for Evolutionary Biology, University of Vienna, Vienna, Austria; Center for Anatomy and Cell Biology, Medical University of Vienna, Vienna, Austria; Natural History Museum Vienna, Central Research Laboratories, Vienna, Austria

**Keywords:** museomics, *Drosophila melanogaster*, phylogenomics, *Wolbachia*, evolutionary history, contamination

## Abstract

*Wolbachia* is one of the most common bacterial endosymbionts, which is frequently found in numerous arthropods and nematode taxa. *Wolbachia* infections can have a strong influence on the evolutionary dynamics of their hosts since these bacteria are reproductive manipulators that affect the fitness and life history of their host species for their own benefit. Host–symbiont interactions with *Wolbachia* are perhaps best studied in the model organism *Drosophila melanogaster*, which is naturally infected with at least 5 different variants among which *w*Mel and *w*MelCS are the most frequent ones. Comparisons of infection types between natural flies and long-term lab stocks have previously indicated that *w*MelCS represents the ancestral type, which was only very recently replaced by the nowadays dominant *w*Mel in most natural populations. In this study, we took advantage of recently sequenced museum specimens of *D. melanogaster* that have been collected 90 to 200 yr ago in Northern Europe to test this hypothesis. Our comparison to contemporary *Wolbachia* samples provides compelling support for the replacement hypothesis. Our analyses show that sequencing data from historic museum specimens and their bycatch are an emerging and unprecedented resource to address fundamental questions about evolutionary dynamics in host–symbiont interactions. However, we also identified contamination with DNA from crickets that resulted in co-contamination with cricket-specific *Wolbachia* in several samples. These results underpin the need for rigorous quality assessments of museomic data sets to account for contamination as a source of error that may strongly influence biological interpretations if it remains undetected.

## Introduction

*Wolbachia pipientis* (Hertig 1936) is a Gram-negative α-proteobacterium of the order Rickettsiales, which represents one of the most common endosymbionts in animals (reviewed in Landmann 2019; Kaur et al. 2021). *Wolbachia* has been detected in approximately 50% of all arthropods and some filarial nematode species (Zug and Hammerstein 2012; Weinert et al. 2015; Lefoulon et al. 2016; Sazama et al. 2017) and can have a substantial impact on the life history and fitness of its host. These effects might be transient (Correa and Ballard 2016) and range from parasitic to mutualistic with different fitness effects on the host (Zug and Hammerstein 2015) depending on the host species, the *Wolbachia* type, and the environment (reviewed in Pietri et al. 2016; Chrostek et al. 2021; Strunov et al. 2022). Moreover, *Wolbachia* can act as a reproductive parasite causing cytoplasmic incompatibility, feminization, male killing, and parthenogenesis (reviewed in Landmann 2019; Kaur et al. 2021). Since *Wolbachia* is maternally transmitted, these reproductive manipulations usually lead to a higher prevalence and increased fitness of infected females in a population (reviewed in Werren et al. 2008). Moreover, by enhancing host fitness and fecundity (Dedeine et al. 2001; Hosokawa et al. 2010; Miller et al. 2010) and by providing protection against RNA viruses (Hedges et al. 2008; Teixeira et al. 2008; Moreira et al. 2009; Osborne et al. 2009), *Wolbachia* can behave as facultative or obligate mutualists (Zug and Hammerstein 2015). *Wolbachia* thus plays an important role in the evolution of its host species (reviewed in Kaur et al. 2021) and can even lead to speciation (Miller et al. 2010).

Currently, *Wolbachia* is classified into several phylogenetic supergroups traditionally subsumed under *W. pipientis* (Bandi et al. 1994; Werren et al. 1995; Lo et al. 2002; Olanratmanee et al. 2021). However, systematics of the genus *Wolbachia* is still under debate, and there is no consensus yet on whether supergroups represent distinct species or just lineages of *W. pipientis* (e.g. Ramírez-Puebla et al. 2015; Lindsey et al. 2016 and references therein). In the following, we thus use *Wolbachia* synonymously for *W. pipientis*. Most *Wolbachia*-infecting insects belong to supergroups A and B (Lo et al. 2002; Gerth et al. 2014). However, due to horizontal transfers across species boundaries, the phylogenetic tree of *Wolbachia* is often incongruent with the species tree of their hosts (Scholz et al. 2020; Gomes et al. 2022). In fact, several insect host species, such as the vinegar fly *Drosophila simulans*, can even carry *Wolbachia* variants that belong to different supergroups, such as type *w*Ri (Hoffmann et al. 1986) and *w*Ha (O’Neill and Karr 1990) from supergroup A and type *w*No from supergroup B (Poinsot and Merçot 1997). However, with the exception of *Drosophila mauritiana*, whose *Wolbachia* symbiont is of supergroup B (Meany et al. 2019), most other species of the genus *Drosophila* have *Wolbachia* types that belong to supergroup A only. The genetic model species *Drosophila melanogaster*, for example, has been found to carry at least 5 distinct *Wolbachia* variants of supergroup A in natural populations (Riegler et al. 2012). A large body of literature documents that specifically the 2 most common natural types *w*Mel and *w*MelCS in *D. melanogaster* and also *w*MelPop, which evolved under laboratory conditions from a *w*MelCS background (Min and Benzer 1997; Woolfit et al. 2013). Both *w*Mel and *w*MelCS are actually further subdivided into several subtypes and form groups of variants (Ilinsky 2013; Ryabinin et al. 2022). In the following, we will use *w*Mel and *w*MelCS synonymously as collective terms for these 2 groups of variants. Both *w*Mel and *w*MelCS can have very distinct fitness effects on their hosts (e.g. Serga et al. 2021; Strunov et al. 2022). For example, *w*MelCS is usually characterized by higher bacterial titers (Chrostek et al. 2013) and can influence reproduction and other life history traits depending on the environment and the host genetic background (Strunov et al. 2022).

The *w*Mel variant is nowadays most common in worldwide *D. melanogaster* populations, whereas *w*MelCS only occurs in a few populations at very low frequencies (Richardson et al. 2012; Chrostek et al. 2013). However, comparisons of *Wolbachia* types in recent populations and long-standing *D. melanogaster* lab strains revealed that old lab stocks, which were often collected almost a century ago, were all dominated by the *w*MelCS variant (Riegler et al. 2005). These findings indicate that *w*Mel only recently replaced the ancestral *w*MelCS variant in worldwide host populations, within the last 50 to 100 yr (Riegler et al. 2005; Early and Clark 2013; Ilinsky 2013). Moreover, since both *Wolbachia* and mitochondria are strictly maternally inherited, their evolutionary histories are tightly intertwined. Several studies showed that mitochondrial (mt) haplotypes are nonrandomly associated with specific *Wolbachia* types (Richardson et al. 2012; Ilinsky 2013; Scholz et al. 2020) and that mt haplotypes can rapidly sweep when they are associated with a putatively adaptive *Wolbachia* variant (Nunes et al. 2008). These studies also found that worldwide *D. melanogaster* populations are dominated by mt haplotypes tightly associated with *w*Mel. This provides further indirect evidence for a pervasive and recent sweep of *w*Mel, although it remains unclear if the dominant mt haplotypes in fly populations predating the sweep were similar to mt types that are currently associated with wMelCS.

Potential biological reasons for such a rapid turnover are also still under debate. Higher titers in *w*MelCS (Chrostek et al. 2013) could result in putatively higher fitness costs to the host, e.g. due to reduced fecundity (see, for example, Serga et al. 2014), which may have facilitated the spread of the low-titer *w*Mel type. Alternatively, threats by RNA viruses may have changed over time, which could have reduced the benefit of higher virus protection by *w*MelCS (Hedges et al. 2008; Teixeira et al. 2008; Moreira et al. 2009; Osborne et al. 2009; Chrostek et al. 2021) and subsequently benefitted the spread of *w*Mel.

However, given that no data of historic samples from this time period were available until now, it remains unclear to which extent the findings in Riegler et al. (2005) reflect the true evolutionary history of both types in natural populations. For example, lab stocks may be prone to contamination in stock centers, which could have biased their infection type if *w*MelCS is more successful than *w*Mel under laboratory conditions. This could have accordingly resulted in an excess of *w*MelCS infections in lab strains and thus confound the interpretation of these data. Moreover, the origin and pervasiveness of the invasion and subsequent replacement are also under debate since residual occurrence of *w*MelCS infections in natural populations has been identified in Europe and North America (Richardson et al. 2012).

In our study, we are for the first time able to empirically address this long-standing question by taking advantage of a recently published genomic data set from *D. melanogaster* museum specimens, which were collected 90 to 200 yr ago in Northern Europe (Shpak et al. 2023). We tested for the presence of *Wolbachia*-specific reads in these data, estimated titer variation and investigated the relatedness to contemporary *Wolbachia* strains. This unique data set, which we complemented with contemporary genomic data from various sources, showed that *D. melanogaster* populations in Northern Europe were indeed dominated by the ancestral *w*MelCS types up to 90 yr ago, which further supports the hypothesis that *w*Mel only recently replaced *w*MelCS in worldwide populations. These findings are further supported by the comparison of historic and contemporary mt haplotypes that revealed that historic *D. melanogaster* samples were dominated by mt haplotypes that are strongly associated with *w*MelCS in historic and recent populations.

## Materials and Methods

In total, 69 genomic data sets were included in the present study (supplementary table S1, Supplementary Material online): Recently published whole genomic Illumina deep sequencing data of 25 historic *D. melanogaster* museum specimens (Shpak et al. 2023) were tested for the presence of *Wolbachia*-specific reads. These samples originated from Sweden, Denmark, and Germany and had been collected between 90 and 200 yr ago. Complementary to these historic samples, we used Oxford Nanopore Technologies (ONT) sequencing to newly sequence genomic DNA of 6 freshly collected isofemale lines from wild populations in Portugal and Finland that were naturally infected with either the *w*Mel or the *w*MelCS *Wolbachia* types and 3 lab stocks that had been artificially infected with *w*Mel, *w*MelCS, and *w*MelPOP (see supplementary table S1, Supplementary Material online). Furthermore, we included 25 raw Illumina sequencing data sets of contemporary *D. melanogaster* specimens, which were either uninfected (*n* = 3) or previously tested positive for infections with *w*Mel (*n* = 20) or *w*MelCS (*n* = 2) (Richardson et al. 2012), for phylogenetic analyses (see supplementary table S1, Supplementary Material online). These samples are part of the *Drosophila* NEXUS data sets (Pool et al. 2012; Lack et al. 2015, 2016) and the DGRP data sets (Mackay et al. 2012), which represent huge collections of single individual sequencing data of natural flies that were mostly collected in Africa and North America, respectively. Since these data sets contained only 2 specimens infected with *w*MelCS, we further included the raw sequencing data sets of 2 lab stocks carrying *w*MelCS (Woolfit et al. 2013; Duarte et al. 2021) that were enriched for bacterial sequences during the library preparation. Complementary to these raw sequencing data, we further obtained 9 RefSeq assemblies (Wu et al. 2004; Singhal and Mohanty 2018; Basting and Bergman 2019; Duarte et al. 2021) for phylogenetic inference based on BUSCO genes—eight of them carrying *w*Mel and one of them a *w*MelCS type (see supplementary table S1, Supplementary Material online). Given the close relationship between *w*Yak (the *Wolbachia* strain of *Drosophila yakuba*) and *w*Mel (Scholz et al. 2020), we used the RefSeq sequence information of the *w*Yak *Wolbachia* type (NZ_VCEF01000001.1) as an outgroup for phylogenetic tree reconstructions.

### DNA Extraction, Library Preparation, and ONT Whole-Genome Sequencing

Prior to whole-genome sequencing of 9 contemporary isofemale lines (supplementary table S1, Supplementary Material online) infected with either *w*Mel, *w*MelCS, or *w*MelPOP by means of ONT technology, we confirmed their infection status and infection type with PCR using *Wolbachia*-type-specific VNTR-141 primers and PCR conditions as described in Riegler et al. (2012) (see supplementary fig. S1, Supplementary Material online). Then, we isolated high molecular weight DNA by following the Monarch T3010 DNA purification kit (NEB, USA) protocol. For each of the isofemale lines, we pooled 25 females prior to DNA extraction to increase the DNA yield, assuming that the amount of residual heterozygosity in the strains is very low. After DNA extraction, the DNA was stored at 8 °C for 7 d in order to ensure relaxation and homogenization of the extracts. Prior to library preparations, the integrity/purity of the extracted DNA (A260/A280 and A260/A230 ratios) was assessed with a BioPhotometer (Eppendorf), and quantification of the DNA yield was measured via Qubit Fluorometer (Thermo Fisher Scientific; dsDNA Broad Range Assay Kit). The fragment length distribution of the extracted DNA was inspected on a TapeStation system (Agilent) and extracted DNA was subjected to library preparation by applying the Native Barcoding Kit 24 Sequencing Kit (SQK-NBD112.24) for genomic DNA from ONT. For library preparation, we strictly followed the manufacturer's protocol using 400 ng of gDNA per sample as input. The protocol included a repair and end-prep step, followed by barcode ligation, and subsequent adapter ligation and cleanup step of the pooled samples. After finishing the library preparation, ∼11 fmol of DNA library was loaded on a Flow Cell (R9, FLO-MIN106D), and sequencing was performed on a MinION Mk1C (ONT) for 72 h. We then performed base calling and demultiplexing using the GPU version of guppy (v.6.2.1; Wick et al. 2019).

### Testing for DNA Contamination

**Fig. 1.**
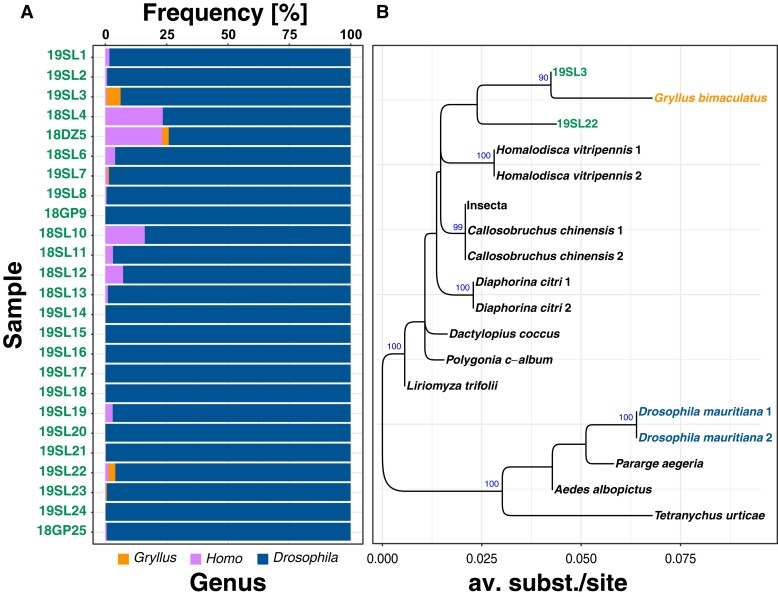
Contamination of historic samples with human and *Gryllus* DNA. Panel A) with stacked barplots showing the frequency of BLAST hits for the 3 most commonly detected genera based on BLAST searches of 100,000 mt reads against the NCBI *nt* database. Panel B) shows a phylogeny based on the *Wolbachia*-specific *wsp* locus for *Wolbachia* samples from supergroup B, an NCBI GenBank sequence from a *Wolbachia* variant specific to *Gryllus bimaculatus* and 2 *Wolbachia* sequences from the historic samples 19SL3 and 19SL22.

Prior to all downstream bioinformatics analyses, we trimmed the raw Illumina reads of historic and contemporary samples using TrimGalore (v.0.6.2; Martin 2011) allowing a minimum base quality of 20. We only retained intact read pairs with a minimum length of 30 bp for further analyses. Then, we tested all historic samples for signals of contamination with foreign DNA. We therefore filtered all trimmed reads for mt sequences using Kraken (v.2.1.2; Wood and Salzberg 2014) with a custom-built database based on the published *D. melanogaster* mt genome (NC_024511.2) and only retained reads that matched the references in the database. Then, we blasted 100,000 randomly drawn filtered mt-specific reads against a local copy of the NCBI *nt* database using the *blastn* algorithm (v.2.12.0; Camacho et al. 2009). We only considered the best match per read passing stringent similarity thresholds (i.e. *e* < 1e^−50^; >99% sequence similarity). Based on the target sequence ID (sseqid) and the NCBI TaxID (staxids), we obtained information on the best hits at the genus level and further analyzed the results in *R* (R Core Team 2019). *Drosophila* was by far the most common BLAST hit in all samples, but we further found signals of human contamination in numerous samples and contamination with mt DNA from crickets of the genus *Gryllus* in 5 samples (19SL3, 18DZ5, 19SL7, 19SL22, and 19SL23; Fig. 1 and supplementary table S2, Supplementary Material online).

Since *Gryllus* is commonly infected with *Wolbachia* from supergroup B (Chafee et al. 2010), we further tested if the *Gryllus* DNA contamination may have resulted in a co-contamination with *Gryllus*-specific *Wolbachia* DNA in the 5 aforementioned samples. To this end, we downloaded raw reads from a representative subset of hosts infected with *Wolbachia* of supergroup B as described in Scholz et al. (2020) (see supplementary table S3, Supplementary Material online). Then, we again used Kraken with a custom-built database consisting of the chromosome-scale genome of *w*Meg, a *Wolbachia* type of the blowfly *Chrysomya megacephala* from supergroup B (RefSeq: NZ_CP021120.1), to enrich the raw sequencing data of the 5 samples and the newly downloaded data for reads specific to supergroup B *Wolbachia*. Finally, we mapped the filtered paired-end reads of each data set against *w*Meg using bwa mem (v.0.7.13; Li, 2013) with default parameters.

Because genome-scale data of *Gryllus*-specific *Wolbachia* are unfortunately not available to date, we downloaded a 593-bp-long sequence of the *Wolbachia wsp* gene of *Gryllus campestris* from GenBank (KC677595.1; Duron 2013)*.* Using exonerate (Slater and Birney 2005) with the model “affine:local,” we aligned the *wsp* sequence against the *w*Meg reference genome to identify the genomic coordinates of the *wsp* fragment along the *w*Meg genome. Then, we used the “consensus” function of samtools (v.1.18; Li et al. 2009) to obtain consensus sequences of the mapped reads at the genomic position of the *wsp* fragment for the aforementioned samples. We only retained historic samples 19SL3 and 19SL22 for further analyses since the other historic samples 18DZ5, 19SL7, and 19SL22 had coverages of <10% at the *wsp* locus and were thus not informative. After aligning all consensus sequences with MAFFT using standard parameters (v.7.487; Katoh and Standley 2013), we reconstructed a maximum likelihood (ML) tree based on the GTR-CAT substitution model from 20 randomly drawn maximum parsimony (MP) starting trees using RaXML (v.2.8.10; Kozlov et al. 2019) and additionally performed 100 rounds of bootstrapping to test for the robustness of nodes.

### Characterization of *Wolbachia* Infections: Relative Titer, and Infection Status

To obtain estimates of *Wolbachia* titers in historic and contemporary samples, we used bwa mem (v.0.7.13; Li, 2013) for Illumina sequencing data or minimap2 (v.2.17; Li, 2018) for ONT sequencing data to map all raw FASTQ reads from each sample against a joint reference sequence, which was constructed from the *D. melanogaster* reference genome v.6 (Hoskins et al. 2015) and additional genome sequences of other common microbial symbionts, including the *w*Mel reference genome (see Kapun et al. 2020 for more details). Using the command *samtools coverage* of the samtools program (v.1.12; Li et al. 2009) in combination with a custom *Python* script (SumReadDepths.py), we calculated average read depths (RDs) for all *Drosophila* chromosomes and symbiont genomes. Using this information, we estimated relative *Wolbachia* titers for a given sample by dividing the average RD at the *Wolbachia* genome by the average RD across all *Drosophila* autosomes (see also Scholz et al. 2020). Based on average RD and the proportion of the *Wolbachia* genome covered by reads, we classified *Drosophila* samples into 3 infection statuses: (i) infected with *Wolbachia* (>50% of the *Wolbachia* genome covered by reads; >10-fold average RD; relative titer > 0.3:1), (ii) uninfected (<15% of the *Wolbachia* reference genome covered by reads; <2-fold average RD; relative titer < 0.01:1), or (iii) with unclear status (15% to 50% of the *Wolbachia* reference genome covered by reads; 2- to 9-fold average RD; relative titer 0.02:1 to 0.2:1). These thresholds are necessarily arbitrary, albeit informed by a strong correlation between RD and coverage in the historic samples (supplementary fig. S2, Supplementary Material online), which indicates a much stronger decay of historic *Wolbachia* genomes compared to contemporary samples.

### *Wolbachia* Classification based on Diagnostic SNPs

First, we filtered all historic and contemporary samples for *Wolbachia*-specific reads. We therefore used Kraken (v.2.1.2; Wood and Salzberg 2014) with a custom-built database that consisted of the published genomes of *w*Mel (RefSeq: AE017196.1), *w*MelCS (RefSeq: NZ_JACSNK000000000.1), and *w*MelPOP (RefSeq: NZ_AQQE00000000.1) and only retained reads that matched the references in the database. For the Illumina sequencing data of historic and contemporary samples downloaded from NCBI SRA, we mapped paired-end reads using bwa mem (v.0.7.13; Li, 2013) with default parameters against the *w*Mel reference genome (RefSeq: AE017196.1). Conversely, we used minimap2 (v.2.17; Li, 2018) with default parameters to map long-fragment reads from ONT sequencing against the reference *w*Mel genome. Raw BAM files were filtered with samtools (v.1.12; Li et al. 2009) to contain mapped reads only (parameter -F 4) and sorted by reference position using the *samtools sort* command. Then, we used the BCFtools (v.1.16; Danecek et al. 2021) command *bcftools mpileup* to synchronize the mapped reads of all samples and called SNPs using *bcftools call* assuming haploidy and stored the types in the VCF file format and only considered biallelic polymorphic positions where the posterior probability of the most likely genotype was >50 and of the alternative genotype was <30. Assuming haploidy for variant calling further reduces the probability that low levels of contamination with foreign *Wolbachia* confound the SNP detection.

When comparing 33 contemporary infected samples with known *Wolbachia* type, we identified 67 SNPs that were fixed for different alleles in the 25 *w*Mel and the 8 *w*MelCS samples, respectively (supplementary table S1, Supplementary Material online). Using these *Wolbachia*-type-specific markers SNPs, we inferred the allelic state at each SNP position in the historic samples that were putatively infected with *Wolbachia* and that were not contaminated with *Gryllus* (see above). We only considered a position diagnostic whenever the RD was ≥2 and the allelic state was unambiguously identified based on a posterior probability as explained above. Two of the samples with uncertain infection status (19SL2 and 18SL6) did not have coverage at any of the diagnostic markers and thus had to be excluded.

### De Novo Assembly and Draft Annotation of the *Wolbachia* Genomes

In the next step, we used raw FASTQ files of each library that were trimmed and enriched for *Wolbachia-*specific reads with Kraken using a custom database consisting of *w*Mel, *w*MelCS, and *w*MelPOP reference genomes as described above for de novo assembly with SPAdes (v.3.15.3; Bankevich et al. 2012; Prjibelski et al. 2020) using default parameters. Raw long-fragment reads from ONT sequencing of contemporary flies were assembled with Flye (v.2.9; Lin et al. 2016; Kolmogorov et al. 2019) using default parameters. Raw assemblies were finally polished in 2 rounds using decona (v.0.1.3; Oosterbroek et al. 2021).

Subsequently, assembly quality was assessed based on common quality statistics, such as numbers of contigs, N50 and N90 with QUAST (v.5.1.0rc1; Mikheenko et al. 2018). We tested for assembly completeness using the BUSCO approach (v.5.2.2; Seppey et al. 2019; Manni et al. 2021), where the proportion of intact, fragmented, and missing benchmarking universal single-copy orthologs (BUSCO) specific to the bacterial order Rickettsiales (rickettsiales_odb10) was evaluated in each assembled genome. In addition, we remapped the raw reads to the assembled contigs using minimap2 (v.2.17; Li, 2018) to assess variation in RD, and we compared all contigs to a local copy of the NCBI *nt* database using *blastn* of the BLAST suite (v.2.12.0; Camacho et al. 2009). After that, we visualized the results of these quality assessments with Blobtools (v.3.0.0; Laetsch and Blaxter 2017).

Finally, we used the published *Wolbachia w*Mel reference genome (RefSeq: AE017196.1) as a backbone to align and orient the raw contigs with *nucmer* of the MUMmer package (v.3.23; Marçais et al. 2018). Based on *show-tiling* of the MUMmer package, we identified the minimum number of unique contigs that span a maximum of the reference backbone. Using 2 custom Python scripts (CombineContigs.py and SetStart.py), we then combined these contigs into a single scaffold and filled the gaps between each pair of consecutive contigs with a string of 10 N's. Moreover, given that the bacterial genome is circular, we anchored the newly assembled scaffolds at the start point of the reference genome and shifted protruding sequences to the end of the scaffolds.

### Phylogenetic Analysis

We employed 2 complementary approaches to explore the evolutionary history of *Wolbachia* based on phylogenetic inference:

#### SNP-Based Phylogenetic Analysis

Using a custom Python script (BCF2Phylip.py), we converted the VCF file generated in chapter 2.4 to the PHYLIP format, only considering biallelic polymorphic positions where the posterior probability of the most likely genotype was >50 and of the alternative genotype was <30. In addition, we only included positions with more than 5-fold RD in each of the samples and removed samples, with more than 50% missing information across all SNPs. We used a total of 279 aligned SNP data to reconstruct a ML tree based on the GTR-CAT substitution model from 20 randomly drawn MP starting trees using RaXML (v.2.8.10; Kozlov et al. 2019) and additionally performed 100 rounds of bootstrapping to test for the robustness of nodes. The final tree was plotted in *R* (v.4.2.2; R Core Team 2019) using the *ggtree* package (Yu et al. 2017). Using a custom Python script (SNPDiff.py), we further counted the number of fixed differences between *w*Mel and *w*MelCS as well as among historic and contemporary *w*MelCS variants and among the 5 subgroups of *w*Mel following the classification from Richardson et al. (2012).

#### Phylogenetic Analysis Based on BUSCO Genes

Complementary to the approach described above, we compared the nucleotide sequences of candidate genes as identified by the BUSCO approach from the de novo assembled genomes of the historic museum specimens and the contemporary samples. We therefore focused on 211 BUSCO genes specific to the bacterial order Rickettsiales, which were identified as complete and which were present in the majority of the assembled genomes in our data set. Using MAFFT with standard parameters (v.7.487; Katoh and Standley 2013), we aligned their sequences separately and subsequently concatenated the alignments with a custom Python script (ConcatenateAlignments.py; final length of concatenated alignment: 178,150 bp). Finally, we manually edited the alignment file in JalView (Waterhouse et al. 2009) to repair local misalignments and reconstructed a ML tree based on the GTR-Gamma substitution model from 20 distinct randomized MP starting trees and additionally performed 100 rounds of bootstrapping to test for the robustness of nodes using RaXML (v.2.8.10; Kozlov et al. 2019). The final tree was plotted in *R* (v.4.2.2; R Core Team 2019) using the *ggtree* package (Yu et al. 2017).

### Comparison to mt Phylogeny

*Wolbachia* and the host mitochondria are both transmitted maternally to the offspring and should thus share a similar evolutionary history (Nunes et al. 2008; Richardson et al. 2012; Ilinsky 2013). This allows testing for genomic signals of horizontal introgression of *Wolbachia* into a host, which would manifest in inconsistencies among the species trees of mitochondria and *Wolbachia*. We therefore employed the SNP-based phylogenetic approach described above also based on mt reads, which we prefiltered by comparing all raw reads against a custom-built Kraken database consisting of the *D. melanogaster* reference mt genome (RefSeq: NC_024511.2). To this end, we included all historic samples irrespective of their infection status. We then performed a phylogenetic analysis with this data set using RaXML as explained above.

In addition, we used a subset of the *Wolbachia* and mt SNP data sets that included the same samples and performed a phylogenetic analysis as described above. Then, we used the function *as.dendrogram* from the *R* package *phylogram* (Wilkinson and Davy 2018) to convert the *Wolbachia* and mt tree files in NEWICK format to ultrametric trees. We first untangled the 2 trees; i.e. we swapped the branches to best fit the order of the samples using the *untangle* function with the “step1side” method of the *dendextend* package (Galili 2015) and then produced a tanglegram using the *tanglegram* function to visualize the relationship between the 2 trees. In addition, we tested for statistically significant congruence among topologies of the trees based on *Wolbachia* and mitochondria using the Congruence Among Distance Matrices (CADM) test as implemented in the *R* package *ape* (Paradis 2006). To this end, we converted the trees to distance matrixes using the ape function “cophenetic” in *R* (R Core Team 2019) and calculated the CADM test with 100,000 permutations.

## Results and Discussion

In this study, we took advantage of a recently published genomic data set consisting of 25 museum samples of *D. melanogaster* collected between 200 and 90 yr ago in Northern Europe (Shpak et al. 2023). Besides testing if museomics of century-old *Drosophila* samples allows to identify historic *Wolbachia* infections, we address several long-standing questions concerning the co-evolution of *D. melanogaster* and *Wolbachia*. In particular, these data for the first time allow to test the hypothesis that the historically dominating *w*MelCS *Wolbachia* type, nowadays found worldwide only in a few populations at low frequencies, was only recently replaced by the *w*Mel type within the last century or whether the *w*Mel type was already present in European populations centuries ago.

### Several Historic Samples Are Contaminated with DNA from Humans and Crickets

Museomic data are particularly prone to contamination not only due to elevated degrees of DNA degradation in old samples caused by the sample age and preservation methods but also due to storage and handling errors (Raxworthy and Smith 2021). Moreover, because yields from extractions of ancient DNA are usually much lower than from fresh samples, contaminations often happen during the extraction process in the laboratory (see, for example, Irestedt et al. 2022; Anderson et al. 2023). Since contamination can substantially confound phylogenetic and population genetic analyses and their interpretation (e.g. Petrou et al. 2019; Goig et al. 2020), we first assessed the type and amount of potential DNA contamination in the historic samples by blasting 100,000 mt reads per sample against the NCBI *nt* database. We identified pronounced contamination with human DNA ranging from 0.1% to 24% in all but 3 samples (Fig. 1A; supplementary table S2, Supplementary Material online). In addition, we detected contamination with DNA from crickets of the genus *Gryllus* in 5 samples that ranged from 0.4% to 5.7%. Since *Gryllus* species are commonly infected with *Wolbachia* from supergroup B (Mandel et al. 2001), we tested if *Gryllus*-specific *Wolbachia* is present in the samples. We found that between 0.3% (19SL3) and 3% (19SL23) of the total reads for a given sample were similar to *Wolbachia* of supergroup B. To test if these reads indeed originate from *Gryllus*-specific *Wolbachia* rather than from the *Drosophila* host, we further employed a phylogenetic analysis based on fragments of the *Wolbachia*-specific *wsp* locus. Besides two of the historic samples 19SL3 and 19SL22, which had sufficient coverage (>10%) at the *wsp* locus, we further included 1 *Wolbachia* sequence of *G. campestris* from the NCBI GenBank (KC677595.1; Duron 2013) and other *Wolbachia* samples of supergroup B that were obtained from Scholz et al. (2020). As shown in Fig. 1B, the 2 historic samples formed a monophyletic cluster with the cricket sequence. We thus conclude that a DNA contamination with *Gryllus* in five of the historic sample resulted in a subsequent co-contamination with *Gryllus*-specific *Wolbachia* at least in the 2 samples 19SL3 and 19SL23 that we were able to include in the phylogenetic reconstruction. Our analysis emphasizes the importance of rigorous testing for potential contaminations in museomic data. Without this prior knowledge, we would have misinterpreted the detected supergroup B *Wolbachia* variants as being native to the *Drosophila* host.

### *Wolbachia* Detection in Historic *Drosophila* Samples

As a next step, we classified the 25 sequenced historic *Drosophila* samples as infected or uninfected based on reference mapping. Similar to Shpak et al. (2023), we found that the average read length of the historic samples after trimming was approximately 50 bp (supplementary table S1, Supplementary Material online). However, we observed that in spite of our rigorous quality filtering, RDs at the *Drosophila* genome were markedly higher in our study compared to the results in Shpak et al. (2023). We assume that this discrepancy is the result of differences in the mapping pipelines that we employed. Specifically, in contrast to Shpak et al. (2023), we used the more recent *D. melanogaster* reference genome v.6 and the *bwa mem* mapping algorithm. An in-depth visual comparison of mapped reads of historic and contemporary samples with IGViewer (v.2.10.2; Robinson et al. 2011) did not reveal any obvious mapping errors. However, we noted that forward and reverse reads were perfectly overlapping in the historic samples, which is expected given the short fragment size of ∼50 bp (Shpak et al. 2023). Given that we only considered intact read pairs, we do not believe that this introduces a bias in our analyses.

**Fig. 2.**
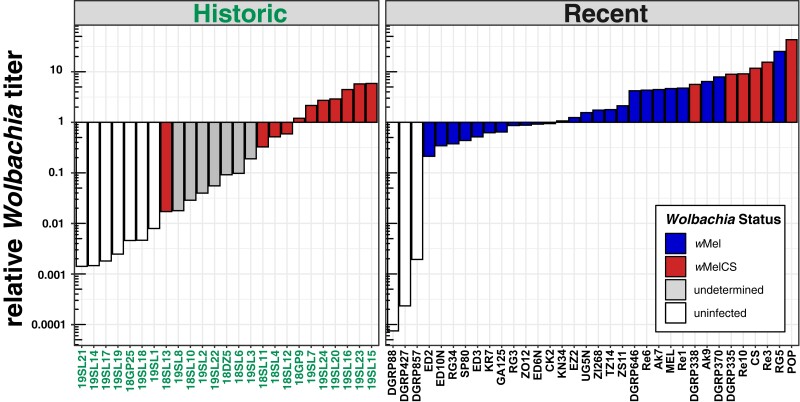
Relative *Wolbachia* titers. Barplots showing the ratio of *Wolbachia*-specific reads to *Drosophila*-specific reads for each library in historic and contemporary samples. The fill color (and hue) depicts the *Wolbachia* type in the recent samples. Here and in all other figures, *w*Mel is shown in blue (dark hue) and both the *w*MelCS and the *w*MelPOP type are shown in red (lintermediate hue). The yet undetermined *Wolbachia* type in historic samples with uncertain infection status is shown in gray (light hue) and uninfected samples are highlighted in white.

We found that absolute *Wolbachia*-specific RD varied between basically 0- to thousand-fold across samples (see supplementary table S1, Supplementary Material online). Similarly, we observed large variations in the length of the reference sequence that was covered by reads. Coverages were ranging from as low as 0.9% to 100%. Since absolute *Wolbachia*-specific RDs are strongly influenced by overall sample-specific RD, we further calculated relative *Wolbachia* titers by dividing *Wolbachia*-specific RD by average *Drosophila*-specific RDs at the 4 autosomal arms (2L, 2R, 3L, and 3R). *Wolbachia* titers relative to the *Drosophila* host in museum samples varied dramatically and ranged from 0.001:1 up to 5:1 (Fig. 2).

Based on these results, we qualitatively classified 10 historic samples as unambiguously infected with *Wolbachia* (see Material and Methods for evaluation criteria). Conversely, we considered 7 historic samples as uninfected. Besides the 2 aforementioned infection types, we identified 8 samples with uncertain infection status, which were characterized by low RD (2- to 9-fold), which covered only parts (15% to 50%) of the reference genome and which had quite low relative titers (0.02:1 to 0.2:1).

Surprisingly, all samples that we classified as uninfected contained even small proportions of reads that mapped to the *Wolbachia* genome. Although we find similarly low or even lower relative titers in uninfected contemporary samples (DGRP427, DGRP857, and DGRP88; Fig. 2), we cannot rule out that low bacterial titers and RDs in putatively uninfected museum samples or samples with uncertain infection status are in fact false negatives. Low titers may have a biological explanation if *Wolbachia* titers in natural samples have been lower 100 to 200 yr ago. Alternatively, the age of the specimen may play a role if in museum samples, *Wolbachia*-specific DNA decays at different rates than *Drosophila*-specific DNA. DNA shearing, depurination, and deamination are major sources of degradation in air-dried museum specimens (Zimmermann et al. 2008; Raxworthy and Smith 2021). With age, the highest decay is observed in dG content (Zimmermann et al. 2008), which might explain the unequal degradation of bacterial and eukaryotic DNA depending on GC content. Moreover, unknown differences in preservation techniques and storage of the historic samples may have further contributed to unequal titer levels.

Both *Wolbachia* strains studied in our manuscript have similar tissue tropism in the host (Albertson et al. 2009). However, *w*Mel is generally characterized by lower titers than *w*MelCS (Chrostek et al. 2013) and may decay faster due to the lower titers than *w*MelCS. While highly unlikely, we thus cannot rule out that we may have failed to detect low-titer *w*Mel variants in uninfected samples. Low titers may also represent an artifact of the DNA extraction or library preparation process, when uninfected flies or samples with uncertain infection status may have inadvertently been cross-contaminated from samples with very high bacterial titers that were processed at the same time (Raxworthy and Smith 2021).

We did not formally test for differences in relative titer due to the rather small and unequal sample sizes and because we did not want to compare different sequencing technologies (Oxford Nanopore vs. Illumina) which may be characterized by different sequencing errors with respect to GC content (see Delahaye and Nicolas 2021). This could lead to biases when comparing the genomes of *D. melanogaster* (GC: 43%; Keightley et al. 2009) and *Wolbachia* (GC: 35.2%; Wu et al. 2004), which substantially differ in GC contents. Nevertheless, we observed qualitative differences in relative titer levels between wMelCS-infected flies from historic and recent samples (see Fig. 2). We, however, assume that these differences are most likely the result of variable DNA degradation rates (as described before) rather than a true biological signal. Moreover, we further observed overall lower relative titer in samples infected with *w*Mel compared to samples with *w*MelCS in the contemporary data set. This is in good agreement with previous data from qPCR-based analyses of bacterial titers, which indicate that relative titers of *w*MelCS are markedly higher and in the order of ≤1:1 for *w*Mel and 1:1 to 10:1 for *w*MelCS types, depending on the age of the flies under laboratory conditions (Chrostek et al. 2013).

**Fig. 3.**
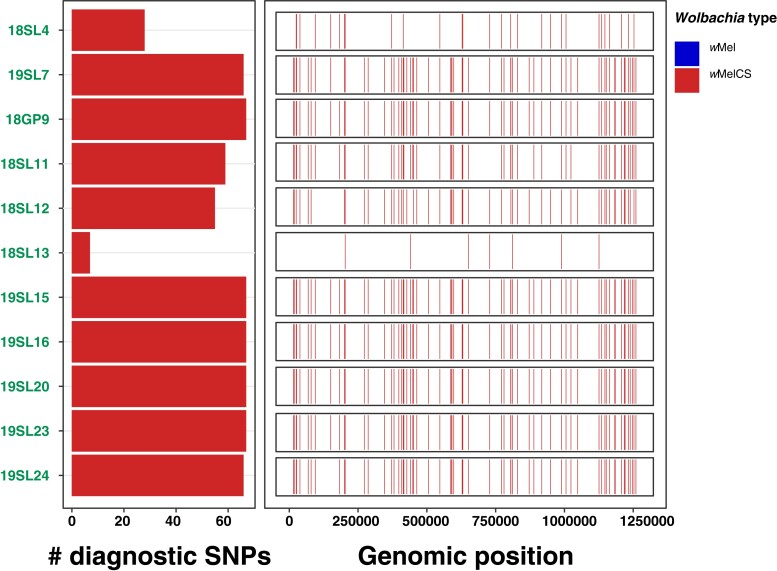
*Wolbachia-*type characterization based on diagnostic marker SNPs. The left barplot shows the allelic states (blue, *w*Mel; red, *w*MelCS) for a maximum of 67 diagnostic *Wolbachia*-type-specific marker SNPs. The size of the bars indicates the number of SNPs with available allelic information for a given sample. The right panel shows the genomic distribution and allelic state of the diagnostic SNPs for a given sample. This figure includes 11 historic samples where allelic information was available for at least 1 diagnostic SNP.

We further tested if the *Wolbachia* variants identified in the historic samples are of *w*Mel and/or of *w*MelCS type. To this end, we took advantage of our genomic data set from recent samples with known *Wolbachia* type and identified 67 diagnostic marker SNPs that were fixed for different allelic states in the 2 types. We were able to investigate 11 historic samples that did not contain foreign arthropod DNA contamination, where at least 3 SNPs contained allelic information due to sufficient RD. All samples of which 10 were previously characterized as infected and 1 was of uncertain infection status based on relative titer were unambiguously identified as infected with *w*MelCS (Fig. 3). While these results strongly support the replacement hypothesis by Riegler et al. (2005), we caution that 10 of these infected samples were collected in Lund, Sweden, albeit in different centuries and only 1 sample (18GP9) was from Passau, Germany. We thus need more data of historic samples from a broader geographic range to confirm that wMelCS was indeed the dominant *Wolbachia* type in historic European populations.

### Phylogenetic Relationships between Historic and Contemporary Wolbachia

**Fig. 4.**
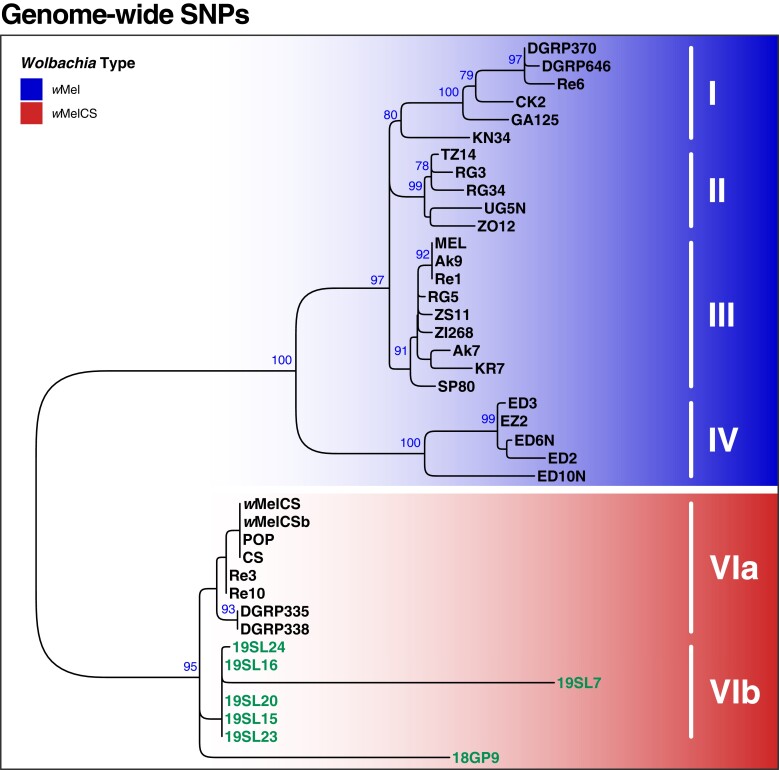
Phylogenetic tree of *Wolbachia* based on 279 genome-wide SNPs. ML tree based on SNP data from 40 samples of historic and contemporary samples that were either infected with *w*Mel (blue) or *w*MelCS (red). Raw reads of each sample were mapped against the *w*Mel reference genome (RefSeq: AE017196.1). Roman numbers indicate subgroups of *D. melanogaster*-specific *Wolbachia* strains as previously defined by Richardson et al. (2012). The 7 historic samples included in this analysis are highlighted in green. Blue numbers indicate bootstrap values > 75% from 100 rounds of bootstrapping.

To better understand the evolutionary history of historic and contemporary *Wolbachia* samples, we mapped raw sequencing data prefiltered for *Wolbachia*-specific reads with Kraken against the *Wolbachia* reference genome and used 279 genome-wide SNPs for phylogenetic inference (Fig. 4). We only included samples in this analysis, which contained sufficient RD at the majority of the SNPs as described in Materials and Methods. Our phylogenetic analyses, which were based on previously available data (including seven of the historic samples) as well as genomic data newly generated by ONT sequencing in this study (9 samples), identified the same 4 phylogenetic clusters (groups I to IV) of *w*Mel types in contemporary samples as previously described in Richardson et al. (2012), which further confirms the robustness of our SNP-based phylogenetic approach. We found that 3% to 14% of all SNPs were fixed differences among these 4 groups, as shown in Table 1. Furthermore, and consistent with previous data, we found that *w*MelCS types form a monophyletic cluster distinct from *w*Mel. The *w*Mel and *w*MelCS clusters were separated by 22% to 25% fixed differences across all SNPs (Table 1), which underlines a pronounced evolutionary distance between the 2 variants. Consistent with our characterization based on diagnostic maker SNPs as described above, we found that all 7 historic samples included in this analysis clustered with the contemporary *w*MelCS samples. The sample 18GP9, which was collected in Germany in the middle of the 19th century formed a separate branch within the *w*MelCS cluster. All other historic samples, which were all collected in 1933 in Sweden, were positioned in a well-supported monophyletic cluster, which we now denote as Group VIb, that was distinct to all contemporary *w*MelCS samples (group VIa; Fig. 4). Consistent with their spatiotemporal relatedness, most samples in this cluster were very similar in terms of genetic variation and only 1% of all SNPs were fixed differences between groups VIa and VIb (Table 1). In contrast, only the branch lengths of samples 19SL7 and 18GP9 were considerably longer than any other terminal branches. Since these 2 samples had relatively high RD (>200) compared to the other 5 samples (see supplementary table S1, Supplementary Material online), we do not assume that the high degree of SNP differences in these samples is likely a technical artifact of the lower sequencing depth resulting in false genotypes but rather a true biological signal or the result of advanced DNA degradation in these 2 samples.

**Table 1:** Fixed SNP difference between the *w*Mel and *w*MelCS groups

SNP counts	*w*Mel					*w*MelCS	
		I	II	III	IV	VIa	VIb
*w*Mel	I	0	14	12	39	70	67
	II	5%	0	7	35	65	63
	III	4%	3%	0	33	63	60
	IV	14%	13%	12%	0	61	62
*w*MelCS	VIa	25%	23%	23%	22%	0	3
	VIb	24%	23%	22%	22%	1%	0

The upper triangle shows absolute numbers of fixed differences and the lower triangle proportion of fixed differences among all SNPs with respect to the *w*Mel and *w*MelCS groups defined in Richardson et al. (2012).

### *Wolbachia* and Mitochondria of *D. melanogaster* Hosts Share a Common Evolutionary History

**Fig. 5.**
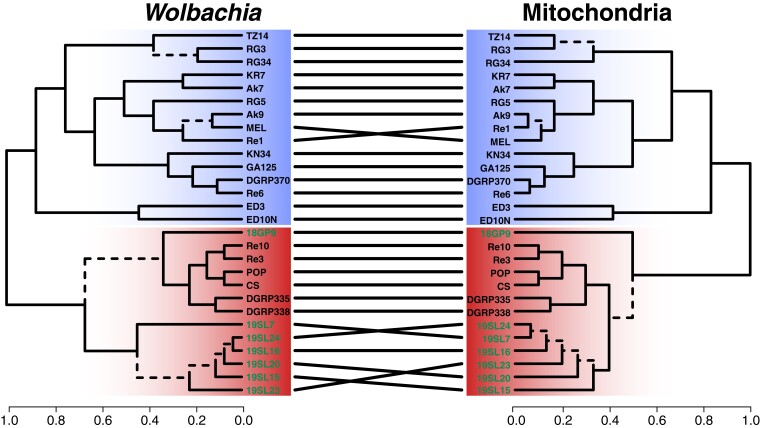
Tanglegram comparing the phylogenetic trees of *Wolbachia* and mitochondria of *D. melanogaster*. Ultrametric trees were generated from SNP-based ML trees for *Wolbachia* and mitochondria of the same historic (highlighted in green) and contemporary samples. Dashed edges indicate topology differences between the trees. Samples of type *w*Mel are highlighted with a blue background and samples of type *w*MelCS with a red background.

Previous analyses have unveiled a close link between the evolutionary histories of *Wolbachia* and mitochondria of the host species (Nunes et al. 2008; Richardson et al. 2012; Ilinsky 2013). Both the organelle and the endosymbiont are jointly transmitted maternally, which results in a strong influence of *Wolbachia* on the distribution of genetic variation in mitochondria (Richardson et al. 2012). This pattern of co-evolution may be only interrupted by sporadic events of horizontal transmission. Here, we tested if we observe similar effects in historic and contemporary *D. melanogaster* samples by applying the same SNP-based phylogenetic analyses to mitochondria-specific reads as described above. A direct comparison of the topologies from mt and *Wolbachia* trees in the form of a tanglegram shown in Fig. 5 and by applying a CADM test reveals a highly significant congruence among the topologies (*P* < 0.0001), which further emphasizes tight links between the evolutionary histories of mitochondria and *Wolbachia*. The only major difference between the 2 trees is the placement of sample 18GP9, which is located at the base of the *w*MelCS cluster in the *Wolbachia* tree but located within the *w*MelCS cluster in the mt tree. Given that the discordance is occurring only on branches with low bootstrap support in either of the 2 trees, we assume that these few inconsistencies are not the result of horizontal transfer but rather of low resolution at the sequence level.

**Fig. 6.**
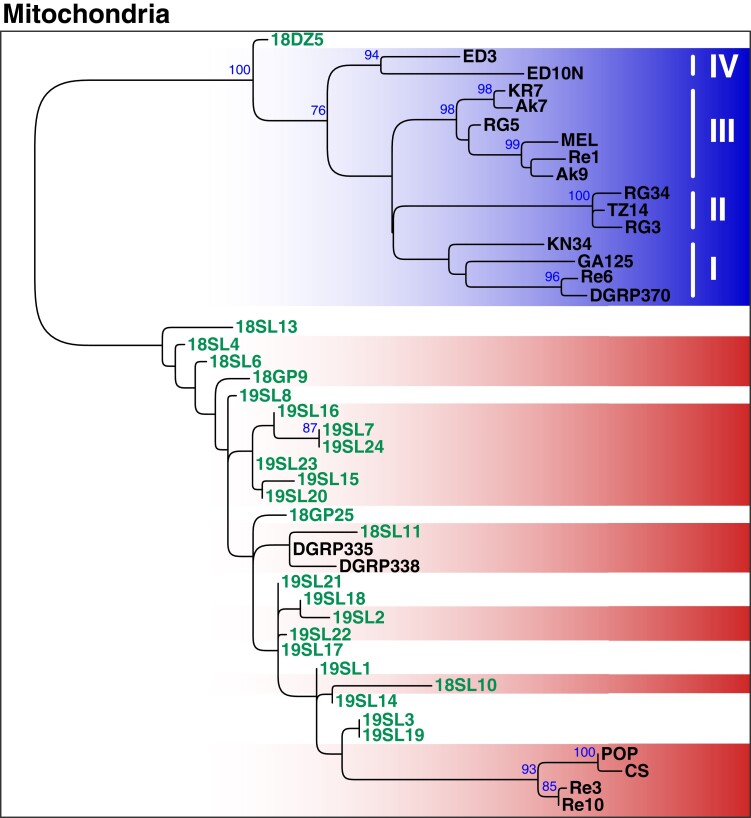
Phylogenetic tree of mitochondria based on 291 genome-wide SNPs. ML tree (mid-point rooted) based on SNP-data from 45 samples of historic and contemporary samples that were either infected with *w*Mel (blue) or *w*MelCS (red) and uninfected specimens are shown in white. The 24 historic samples that were included in this analysis are highlighted in green. Roman numbers indicate clusters that correspond to subgroups of *D. melanogaster*-specific *Wolbachia* strains as previously defined by Richardson et al. (2012). Blue numbers indicate bootstrap values > 75% from 100 rounds of bootstrapping.

Consistent with our expectations and with previous data (Nunes et al. 2008; Richardson et al. 2012; Ilinsky 2013; Scholz et al. 2020), we found that mt samples clustered according to their *Wolbachia* type; i.e. historic and contemporary *w*MelCS-infected samples formed a monophyletic cluster that was distinct from the contemporary *w*Mel samples, which were subdivided into 4 monophyletic clusters that correspond to the *w*Mel groups previously defined in Richardson et al. (2012) (Fig. 6). Given that the 5 samples that showed low levels of contamination with cricket DNA (19SL3, 18DZ5, 19SL7, 19SL22, and 19SL22) did not cluster together, we assume that the mt phylogenetic pattern is not confounded by contamination. Curiously, we found that most of the uninfected historic mt samples were also located within the *w*MelCS cluster. This provides further evidence that modern mt haplotypes that are nowadays associated with *w*Mel were absent or rather rare in Northern Europe, or at least in the geographic area covered by our historic data set, 100 to 200 yr ago. Consistent with this hypothesis, we only found 1 sample (18DZ5) among the 25 historic samples, which was of a *w*Mel-specific mt haplotype. Our analyses of mt haplotype variation in historic samples thus further support a recent sweep of *w*Mel in worldwide populations. However, more data from other historic samples in Europe and North America are needed to narrow down the putative origin of the *w*Mel invasion and to better understand the expansion route of the sweep that led to a turnover not only in *Wolbachia* but also in the associated mt haplotypes.

### De Novo Genome Assemblies of Historic and Contemporary *Wolbachia* Genomes

Finally, we further used the sequencing data of the 10 historic samples, which we classified as infected, and of the 9 newly sequenced contemporary samples for de novo genome assembly. Assembly quality, which we assessed based on the BUSCO approach and on general assembly statistics, such as total number of contigs, cumulative contig lengths, length of the largest contig, N50 and N90, varied highly among the different data sets (supplementary table S4, Supplementary Material online). For five of the historic samples (19SL15, 19SL16, 19SL20, 19SL23, and 19SL24), we were able to produce de novo assemblies where the cumulative lengths of *Wolbachia*-specific contigs were within the range of the expected total genome size (i.e. approximately 1.26 Mb). In contrast, cumulative contig lengths for the other samples were markedly shorter and ranged from 26 to 590 kb in spite of RD > 10-fold. This suggests elevated levels of sequence degradation in these samples, which is further indicated by N50 values < 100 bp and GC ratios, which are markedly higher than the *w*Mel-specific GC ratio of 35.2%.

**Fig. 7.**
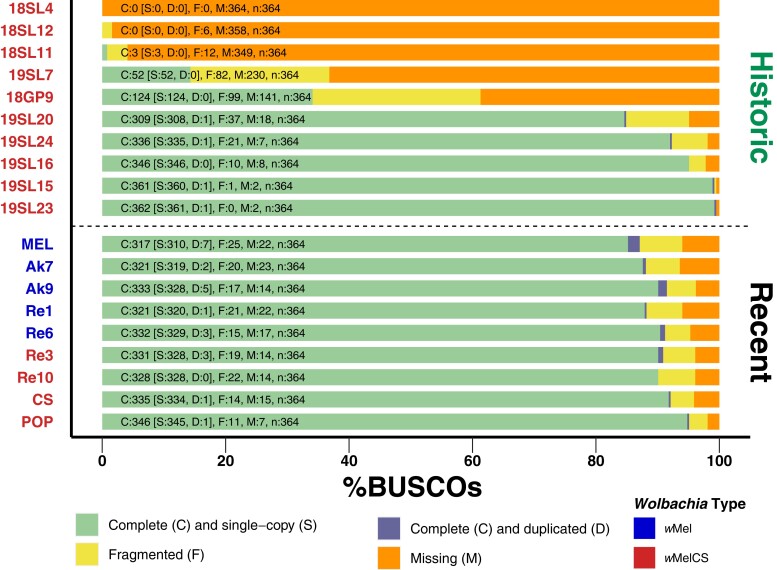
BUSCO scores of de novo assemblies. Stacked barplots showing the proportions of complete, complete and duplicated, fragmented, and missing BUSCO genes of the order Rickettsiales identified in each of the assembled genomes. Historic samples are shown above the dashed lines and 9 newly sequenced specimens are shown below the line. The color of the label names indicates that they are either of type *w*Mel (blue) or *w*MelCS (red).

Consistent with the variable data quality of the investigated historic samples, we found large differences in the proportion of complete BUSCO sequences for the different assemblies (Fig. 7). The 5 high-quality assemblies were characterized by >80% complete and <10% missing BUSCO genes. Conversely, BUSCO results for the other 5 samples ranged from a complete absence of even fragmented BUSCO genes (18SL4) to <40% complete BUSCO (18GP9). Pronounced differences in assembly quality are mostly determined by RD but may also be influenced by differences in the DNA quality, i.e. due to DNA contamination and degradation. This would also be consistent with longer branches of samples 19SL7 and 18GP9 in the SNP-based phylogenetic analysis (Fig. 4), which suggest a pronounced number of private SNPs in these 2 samples.

**Fig. 8.**
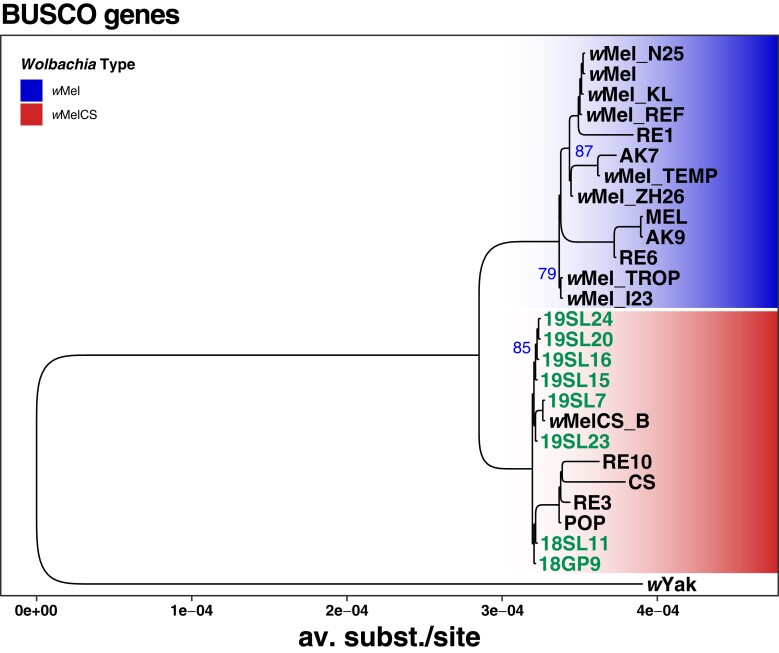
Phylogenetic tree of *Wolbachia* based on 211 aligned and concatenated BUSCO genes. ML tree based on concatenated sequence alignments of 211 genes from 27 historic and contemporary samples that were either infected with *w*Mel (blue) or *w*MelCS (red) and *w*Yak as an outgroup. The 8 historic samples that were included in this analysis are highlighted in green. Blue numbers indicate bootstrap values > 75% from 100 rounds of bootstrapping.

Complementary to the SNP-based phylogenetic analysis, described above, we further aligned 211 BUSCO genes that were complete and present in most of the de novo assembled genomes of historic samples, the newly sequenced contemporary samples and in the RefSeq data sets included in our analyses. In addition, we included the *w*Yak reference genome as an outgroup and reconstructed a ML tree based on the concatenated gene alignments, which yielded a total length of 178,150 bp. The resulting tree (Fig. 8), which includes 5 historic samples that had sufficient RD and data quality to assemble draft genomes, qualitatively confirms our previous findings (Fig. 4). Notably, all 5 historic samples cluster with the contemporary *w*MelCS samples, which provide further support that they were also of *w*MelCS type. While qualitatively similar to the SNP-based phylogeny in Fig. 4, we find that the terminal branch lengths were markedly shorter in the tree based on BUSCO genes compared to the SNP-based phylogeny. While the SNPs that we used for the former approach were distributed genome-wide, we restricted the present phylogenetic reconstruction on BUSCO genes, which represent, per definition, highly conserved genomic regions since they are supposedly occurring in all bacteria of the order Rickettsiales (Seppey et al. 2019; Manni et al. 2021). The short branch lengths in the resulting phylogeny may thus reflect strong purifying selection at these loci. While widely used for phylogenetic reconstructions of distantly related taxa (e.g. Sahbou et al. 2022; Timilsena et al. 2022; Palandačić et al. 2023), this approach may have only limited power to resolve phylogenetic signals of closely related taxa such as samples from the same *Wolbachia* variants.

## Conclusions

In summary, we, for the first time, identified and characterized historic *Wolbachia* infections in *D. melanogaster* samples that were collected between 90 and more than 200 yr ago using publicly available high-quality shotgun sequencing data. Our comparison of historic and contemporary *Wolbachia* samples provides unique empirical evidence that *Wolbachia* infections in Northern European *D. melanogaster* populations were dominated by *w*MelCS-type bacteria. These findings strongly support the hypothesis put forward in Riegler et al. (2005) that the ancestral *w*MelCS type was recently replaced by the nowadays common *w*Mel type. Moreover, these findings are complemented and further supported by the highly similar evolutionary patterns of mt haplotypes. Our analysis shows that historic *D. melanogaster* samples were dominated by mt types that are strongly associated with *w*MelCS in historic and recent populations and that *w*Mel-associated mt haplotypes were very rare. While our evidence is only based on eleven samples that were all characterized as *w*MelCS, these samples were collected across one century in Northern Europe and thus span a broad temporal range. Nevertheless, given that geographic sampling of the full data set is limited to individuals from Lund in Sweden, one sample in Denmark and one sample in Germany, we cannot rule out that *w*Mel already occurred at lower frequencies in the sampled populations or elsewhere in Europe at the time point they were collected. Complementary to several recent studies, which investigated *Wolbachia* evolution on a broad taxonomic (Wu et al. 2004; Scholz et al. 2020) and geographic scale (Richardson et al. 2012; Early and Clark 2013; Basting and Bergman 2019; Scholz et al. 2020), our analyses provide novel insights into the evolutionary history of *Wolbachia* infections in *D. melanogaster* on a broad temporal scale. We are convinced that the unique resource provided by Shpak et al. (2023) represents a seminal data source and an important proof of concept. Their work and spin-off projects like this study or the study by Scarpa et al. (2023), who used this data set to study the evolutionary history of transposable elements in *D. melanogaster*, will stimulate further quantitative research based on deep sequencing of museum samples (“museomics”). Such new data sets will enable studies to comprehensively quantify and investigate evolutionary change by comparing historic to contemporary genomic data in model and nonmodel organisms preserved in museum collections. However, given the low amounts of DNA and the high levels of degradation, museomic data sets are also particularly prone to contamination and thus require rigorous and novel approaches to test for data quality. This is illustrated by contaminations with human and cricket DNA resulting in co-contaminations with cricket-specific *Wolbachia* that we detected in several samples of the historic data set. Our findings demonstrate the need for thorough quality assessments to avoid drawing false biological conclusions.

## Supplementary Material

msad258_Supplementary_Data

## Data Availability

The full analysis pipeline for this project can be found online (https://sandbox.zenodo.org/doi/10.5072/zenodo.4174). The raw reads from ONT sequencing are deposited at the SRA Archive under the project number PRJNA987350, and the fully assembled genomes and their assembly quality stats are available from the data repository of the Natural History Museum of Vienna (https://doi.org/10.57756/ddvbqk).
