# Supplementary material for: Historic Museum Samples Provide Evidence for a Recent Replacement of Wolbachia Types in European Drosophila melanogaster
Source: Mol Biol Evol. 2023 Nov 23;40(12):msad258. doi: 10.1093/molbev/msad258 (PMC10701101; doi:10.1093/molbev/msad258)
Supplement: msad258_Supplementary_Data [file msad258_supplementary_data.zip › Strunov_et_al_WolbEvolHist_2023_Supplement.docx]

## Historic museum samples provide evidence for a recent replacement of *Wolbachia* types in European *Drosophila melanogaster*.

Anton Strunov^1^, Sandra Kirchner^2^, Julia Schindelar^2^, Luise Kruckenhauser^2,3^, Elisabeth Haring^2,3^, Martin Kapun^1,2,*^

^1^ Medical University of Vienna, Center for Anatomy and Cell Biology

^2^ Natural History Museum Vienna, Central Research Laboratories

^3^ University of Vienna, Department for Evolutionary Biology

^*^ Corresponding Author: Martin Kapun [martin.kapun@meduniwien.ac.at](mailto:martin.kapun@meduniwien.ac.at)


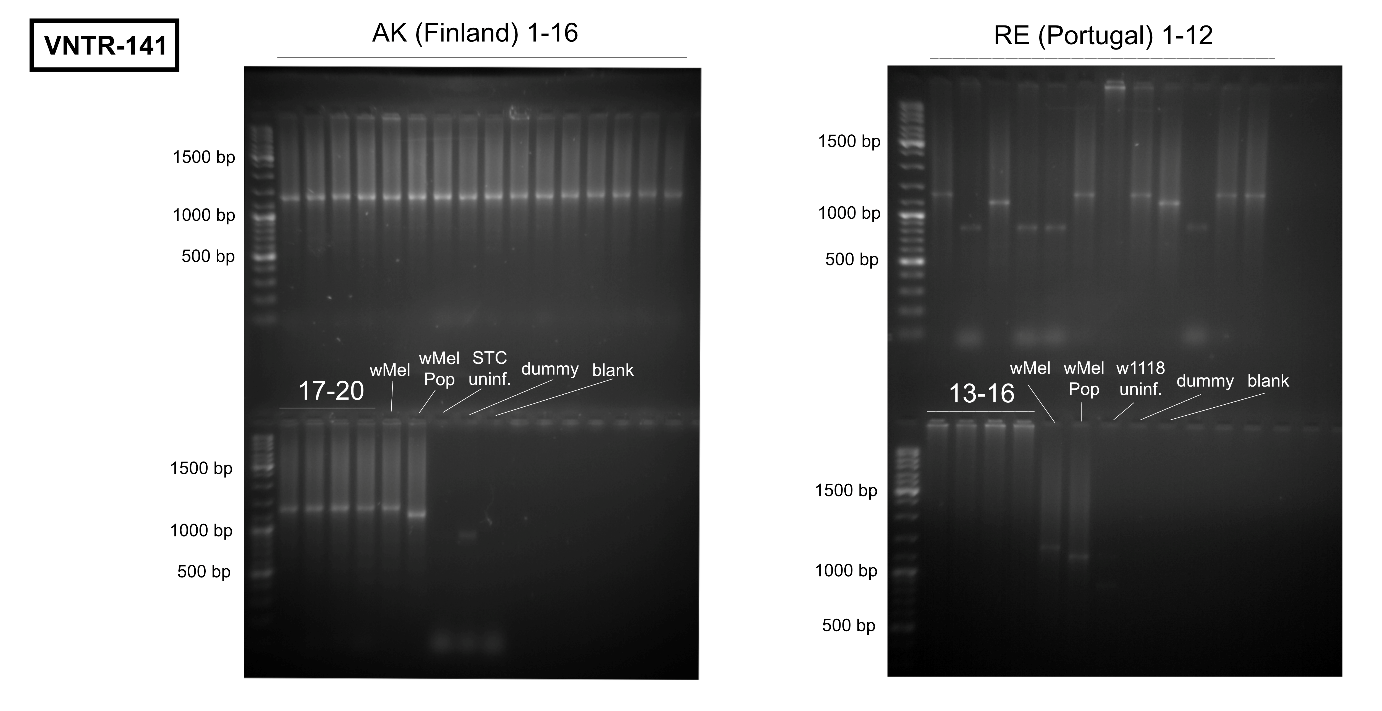


**Supplementary figure S1.** PCR gel-electrophoresis plots of isofemale lines from Finland (AK) and Portugal (RE) that were amplified using the VNTR141 marker from Riegler et al. (2012)


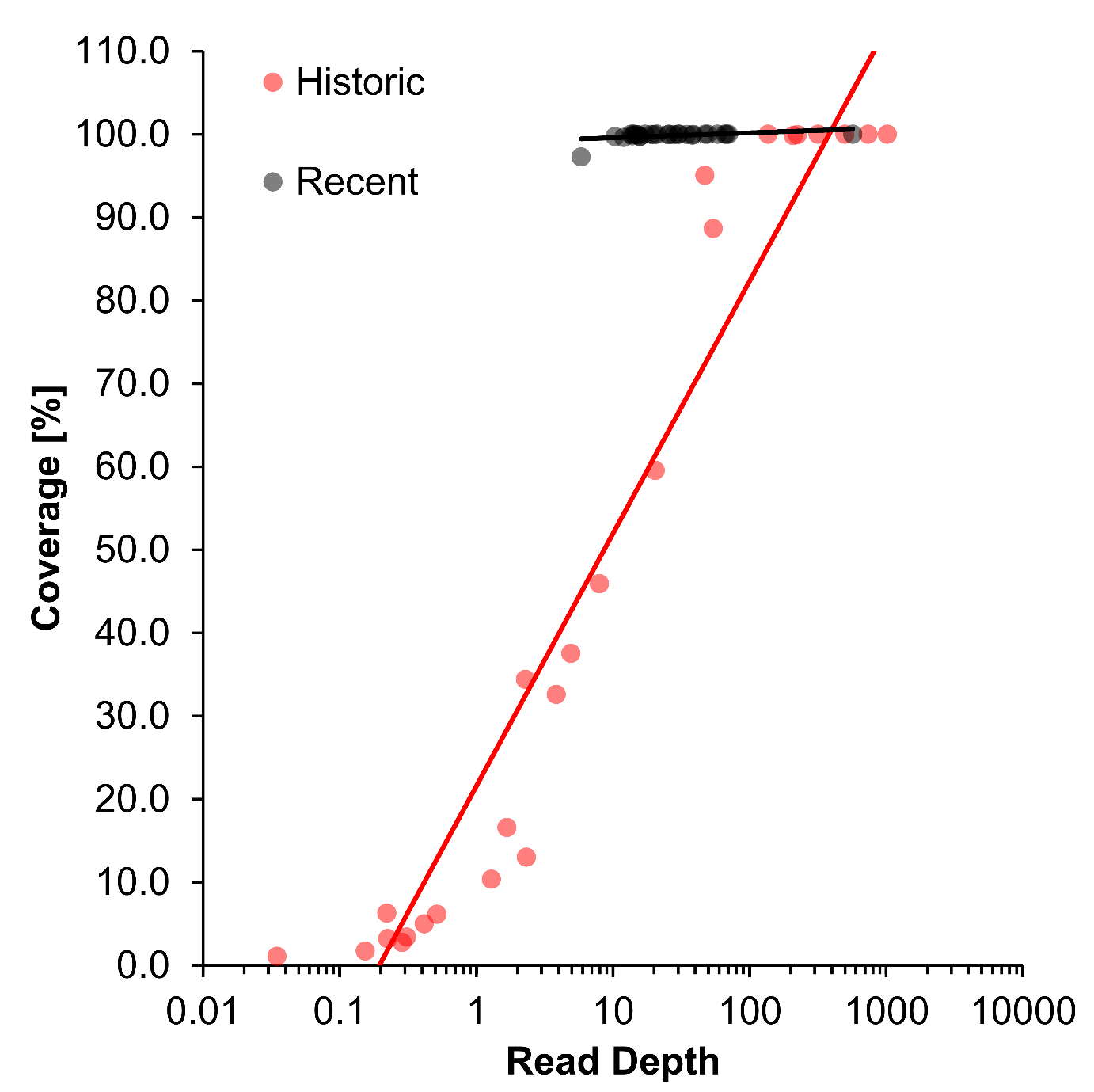


**Supplementary figure S2.** Scatterplot showing the correlation between Read Depth and Genome Coverage for historic (red) and contemporary (black) samples. Note the much steeper regression line for historic samples, which may indicate a stronger decay of historic *Wolbachia* genomes resulting in lower genome coverage if the read depth is not deep enough.
